# Supplementary material for: IL-13Rα2 Regulates the IL-13/IFN-γ Balance during Innate Lymphoid Cell and Dendritic Cell Responses to Pox Viral Vector-Based Vaccination
Source: Vaccines (Basel). 2021 May 1;9(5):440. doi: 10.3390/vaccines9050440 (PMC8147251; doi:10.3390/vaccines9050440)
Supplement: Supplementary file 1 [file vaccines-09-00440-s001.zip › vaccines-1152790-supplementary.pdf]

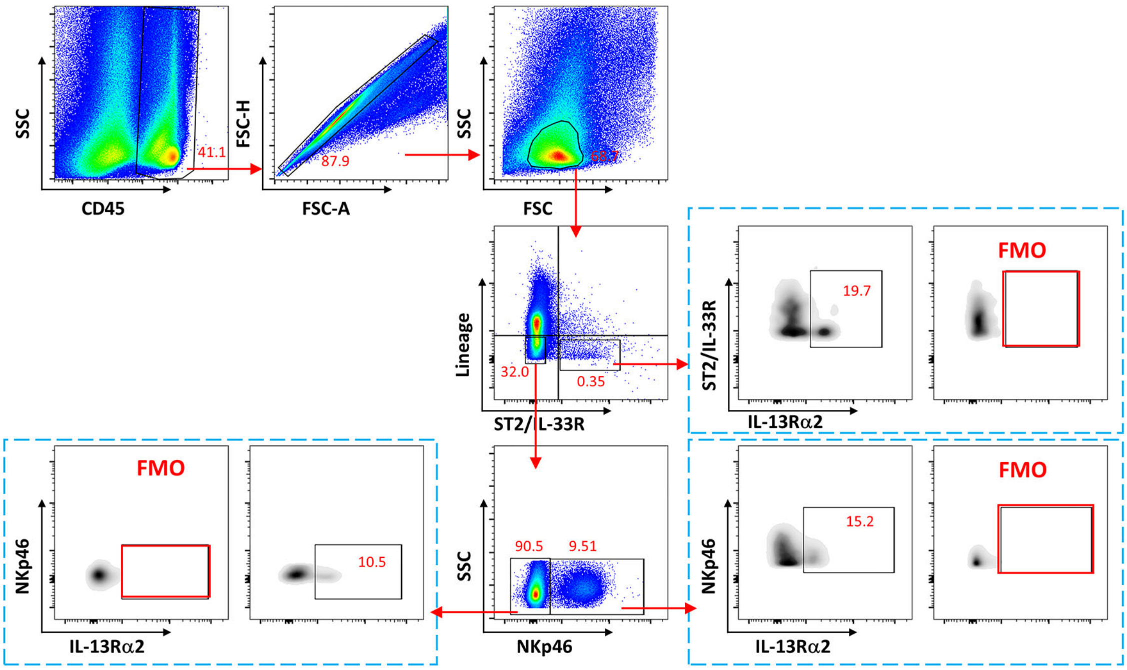

Fig S1. Gating strategy used to assess ILC subsets and their cytokine and IL-13/IL-4 receptor expression. Lung ILCs from BALB/c mice were evaluated 24 h post FPV-HIV immunization as per described in Materials and Methods. ILC2 were gated as $CD45^{+}$ FSC^{low} SSC^{low} $lineage^{-}$ IL-$33R/ST2^{+}$ cells. ILC1/ILC3 were identified as $CD45^{+}$ FSC^{low} SSC^{low} $lineage^{-}$ IL-$33R/ST2^{-}$ $NKp46^{+/-}$ ILCs. γC, IL-4Rα, IL-13Rα1, and IL-13Rα2 expression on different ILC subsets were assessed based on FMO controls. The FACS represent the gating strategy used to assess IL-13Rα2 expression on ILC2 and $NKp46^{+/-}$ ILC1/ILC3.

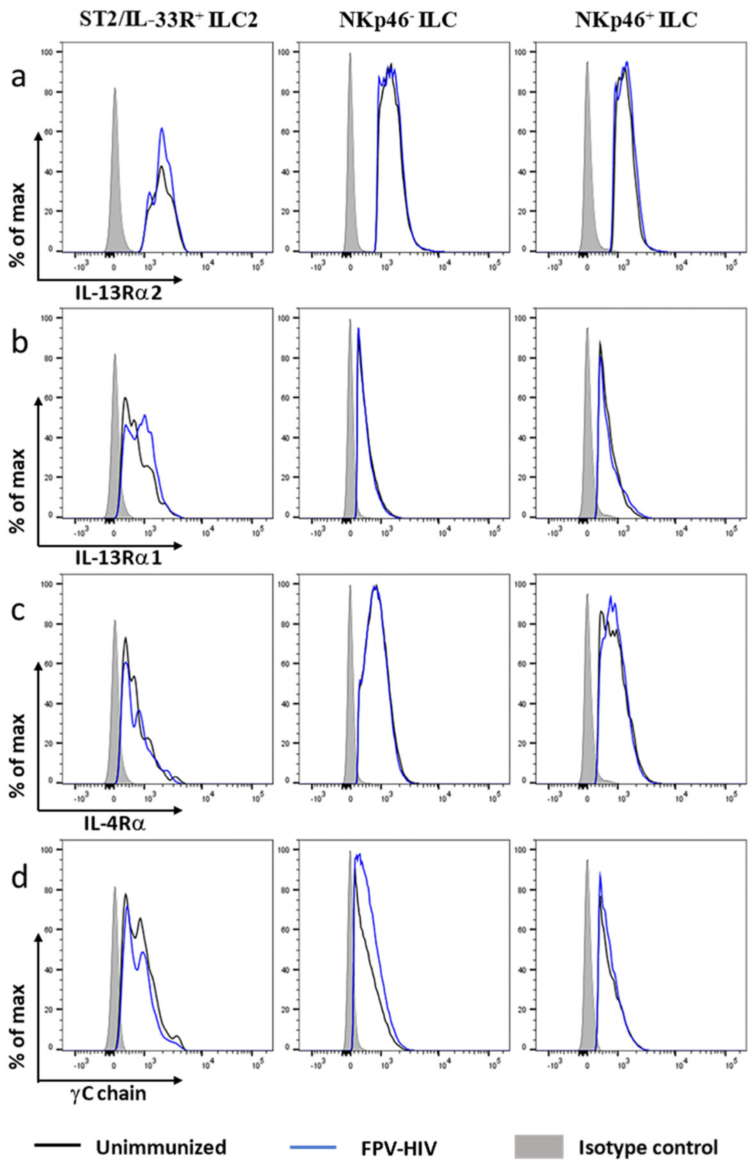

Fig S2. Evaluation of type I (γC chain and IL-4Rα) and type II (IL-4Rα and IL-13Rα1) IL-4 receptor complexes and IL-13Rα2 expression on ILC following intranasal rFPV vaccination. WT BALB/c mice (n = 4) were immunized intranasally with unadjuvanted FPV-HIV vaccine. Using the flow cytometry gating strategy indicated above (Fig S1), ILC2s were defined as $CD45^{+}$ FSC^{low} SSC^{low} $lineage^{-}$ IL-33R/$ST2^{+}$ cells, ILC1/ILC3 were identified as $CD45^{+}$ FSC^{low} SSC^{low} $lineage^{-}$ IL-33R/$ST2^{-}$ $NKp46^{+/-}$ ILCs. The histogram plots indicate the expression density as mean fluorescent intensity (MFI) of IL-13Rα2 **(a)**, IL-13Rα1 **(b)**, IL-4Rα **(c)**, and γC chain **(d)** on different ILC subsets in unimmunized WT BALB/c mice (black line) and 24 h post intranasal unadjuvanted FPV-HIV vaccinated WT BALB/c mice (blue line) compared to isotype control.

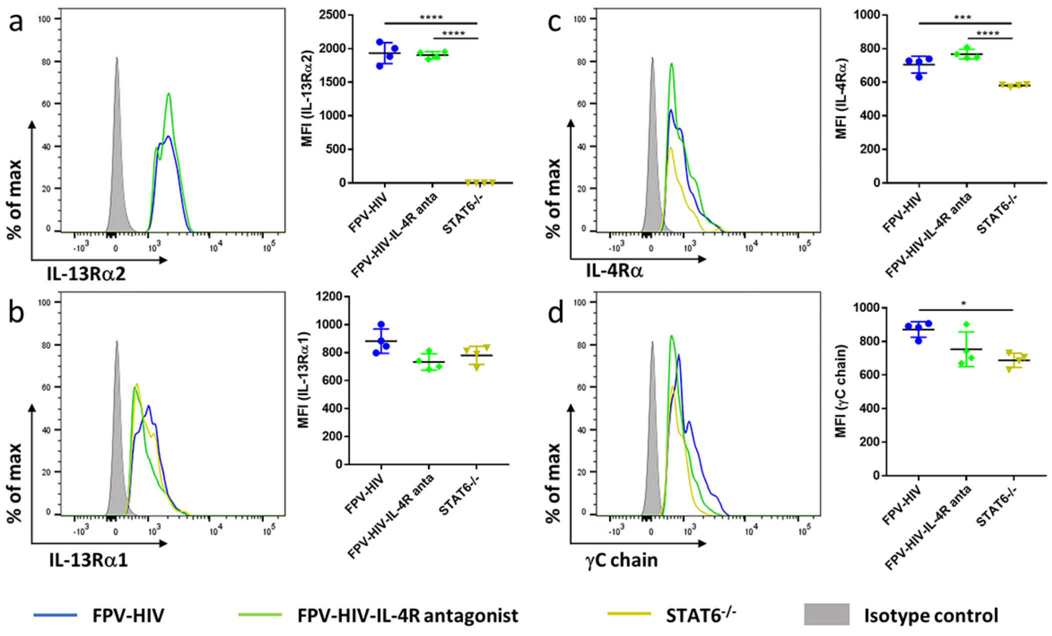

Fig S3. IL-13Rα2, IL-13Rα1, IL-4Rα, and γC receptor densities on ILC2 following rFPV vaccination – (permanent vs transient inhibition of STAT6 signalling). WT BALB/c mice and STAT6$^{-/-}$ mice on BALB/c background (n = 4) were immunized intranasally with FPV-HIV-IL-4R antagonist adjuvanted or FPV-HIV unadjuvanted vaccines respectively. Lung ILC2 were gated as CD45^{+} FSC^{low} SSC^{low} lineage^{-} IL-33R/ST2^{+} cells. The histogram plots show cell surface expression of IL-13Rα2 **(a)**, IL-13Rα1 **(b)**, IL-4Rα **(c)**, and γC chain **(d)** on ILC2s as MFI, [WT BALB/c mice given unadjuvanted FPV-HIV (blue lines) or FPV-HIV-IL-4R antagonist (green lines), and STAT6$^{-/-}$ mice given unadjuvanted FPV-HIV (yellow lines)]. Graphs represent the mean fluorescence intensity of each receptor. The error bars represent the mean and standard deviation (s.d.). The p-values were calculated using GraphPad Prism software (version 6.05 for Windows). * = p<0.05, ** = p<0.01, *** = p<0.001, **** = p<0.0001. For each group experiments were repeated minimum three times.

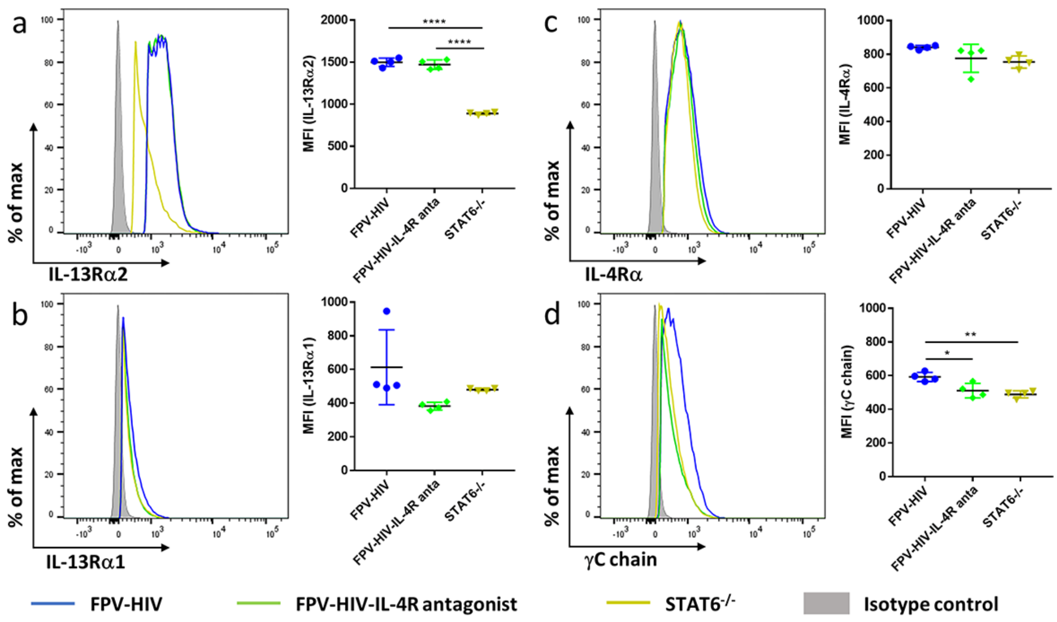

Fig S4. IL-13Rα2, IL-13Rα1, IL-4Rα, and γC receptor densities on lineage⁻ IL-33R/ST2⁻ NKp46⁻ ILC1/ILC3 following rFPV vaccination - (permanent vs transient inhibition of STAT6 signalling). WT BALB/c mice and STAT6$^{-/-}$ mice on BALB/c background (n = 4) were immunized intranasally with FPV-HIV-IL-4R antagonist adjuvanted or FPV-HIV unadjuvanted vaccines respectively. Lung NKp46^{-} ILC1/ILC3 were gated as CD45^{+} FSC^{low} SSC^{low} lineage^{-} IL-33R/ST2^{-} NKp46^{-} cells. The histogram plots in each panel show cell surface expression of IL-13Rα2 **(a)**, IL-13Rα1 **(b)**, IL-4Rα **(c)**, and γC chain **(d)** on lung NKp46^{-} ILC1/ILC3, [WT BALB/c mice given unadjuvanted FPV-HIV (blue lines) or FPV-HIV-IL-4R antagonist (green lines), and STAT6$^{-/-}$ mice given unadjuvanted FPV-HIV (yellow lines)]. Graphs represent the mean fluorescence intensity of each receptor. The error bars represent the mean and standard deviation (s.d.). The p-values were calculated using GraphPad Prism software (version 6.05 for Windows). * = p<0.05, ** = p<0.01, *** = p<0.001, **** = p<0.0001. For each group experiments were repeated minimum three times.

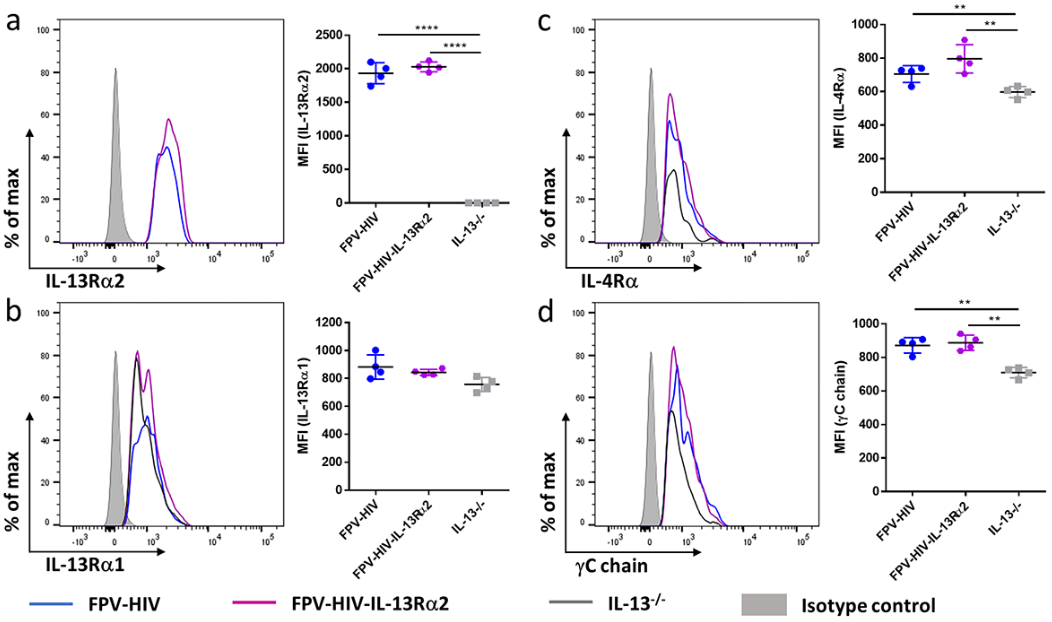

Fig S5. IL-13Rα2, IL-13Rα1, IL-4Rα, and γC receptor densities on ILC2 following unadjuvanted and IL-13Rα2 adjuvanted vaccination - (permanent vs transient inhibition of IL-13 signalling). WT BALB/c mice and IL-13$^{-/-}$ mice on BALB/c background (n = 4) were immunized intranasally with FPV-HIV-IL-13Rα2 adjuvanted or FPV-HIV unadjuvanted vaccines respectively. Lung ILC2 were gated as CD45^{+} FSC^{low} SSC^{low} lineage^{-} IL-33R/ST2^{+} cells. The histogram plots in each panel show cell surface expression of IL-13Rα2 **(a)**, IL-13Rα1 **(b)**, IL-4Rα **(c)**, and γC chain **(d)** on lung ILC2 [WT BALB/c mice given unadjuvanted FPV-HIV (blue lines) or FPV-HIV-IL-13Rα2 adjuvanted (purple lines), and IL-13$^{-/-}$ mice given unadjuvanted FPV-HIV (grey lines)]. Graphs represent the mean fluorescence intensity of each receptor. The error bars represent the mean and standard deviation (s.d.). The p-values were calculated using GraphPad Prism software (version 6.05 for Windows). * = $p<0.05$, ** = $p<0.01$, *** = $p<0.001$, **** = $p<0.0001$. For each group experiments were repeated minimum three times.

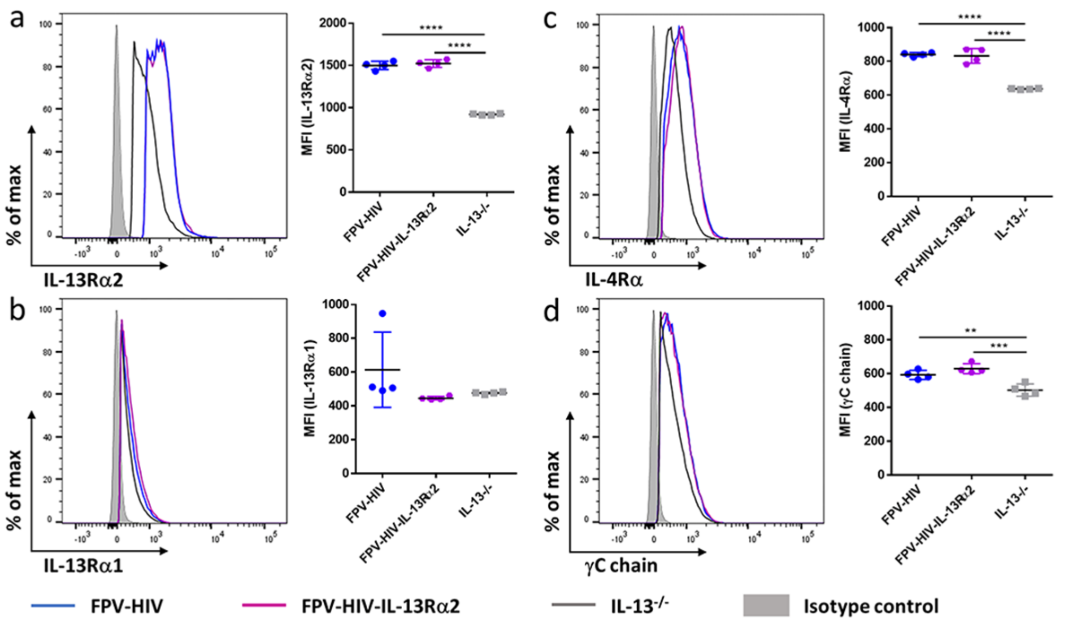

Fig S6. IL-13Rα2, IL-13Rα1, IL-4Rα, and γC receptor densities on lineage- IL-33R/ST2- NKp46- ILC1/ILC3 following unadjuvanted and IL-13Rα2 adjuvanted vaccination - (permanent vs transient inhibition of IL-13 signalling). WT BALB/c mice and IL-13$^{-/-}$ mice on BALB/c background (n = 4) were immunized intranasally with FPV-HIV-IL-13Rα2 adjuvanted or FPV-HIV unadjuvanted vaccines respectively. Lung NKp46^{-} ILC1/ILC3 were gated as CD45^{+} FSC^{low} SSC^{low} lineage^{-} IL-33R/ST2^{-} NKp46^{-} cells. The histogram plots in each panel show cell surface expression of IL-13Rα2 **(a)**, IL-13Rα1 **(b)**, IL-4Rα **(c)**, and γC chain **(d)**, on lung NKp46^{-} ILC1 [WT BALB/c mice given unadjuvanted FPV-HIV (blue lines) or FPV-HIV-IL-13Rα2 adjuvanted (purple line) IL-13$^{-/-}$ mice given unadjuvanted FPV-HIV (grey lines)]. Graph represents the mean fluorescence intensity (MFI) of each receptor. The error bars represent the mean and standard deviation (s.d.). The p-values were calculated using GraphPad Prism software (version 6.05 for Windows). * = p<0.05, ** = p<0.01, *** = p<0.001, **** = p<0.0001. For each group experiments were repeated minimum three times.

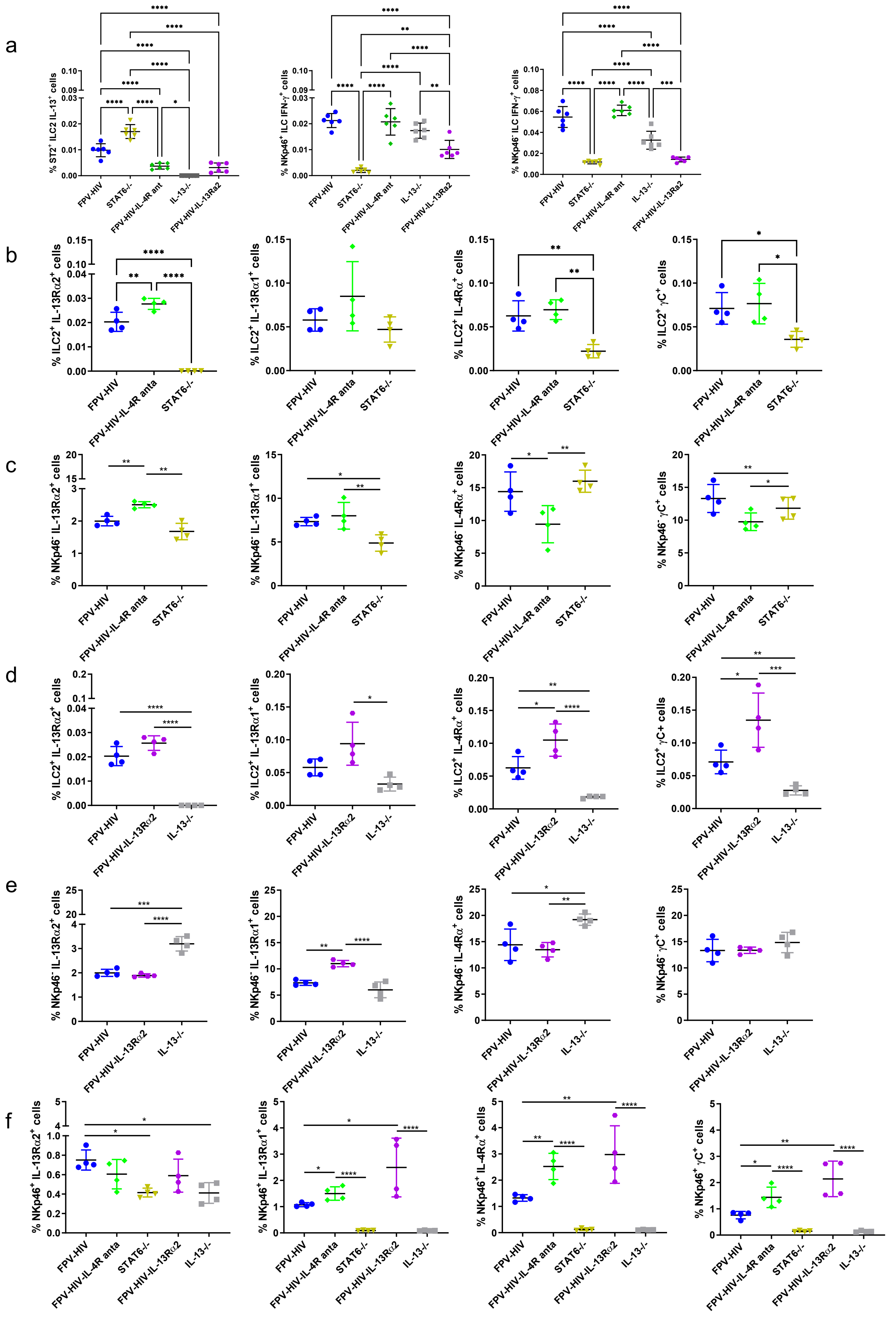

Fig S7. Results from Fig 1 to 6 presented in the form of percentage out of CD45^{+} cells. Panels **(a)** to **(f)** are showing the same results in Fig 1 to 6 respectfully, presented in the form of percentage out of CD45^{+} cells.

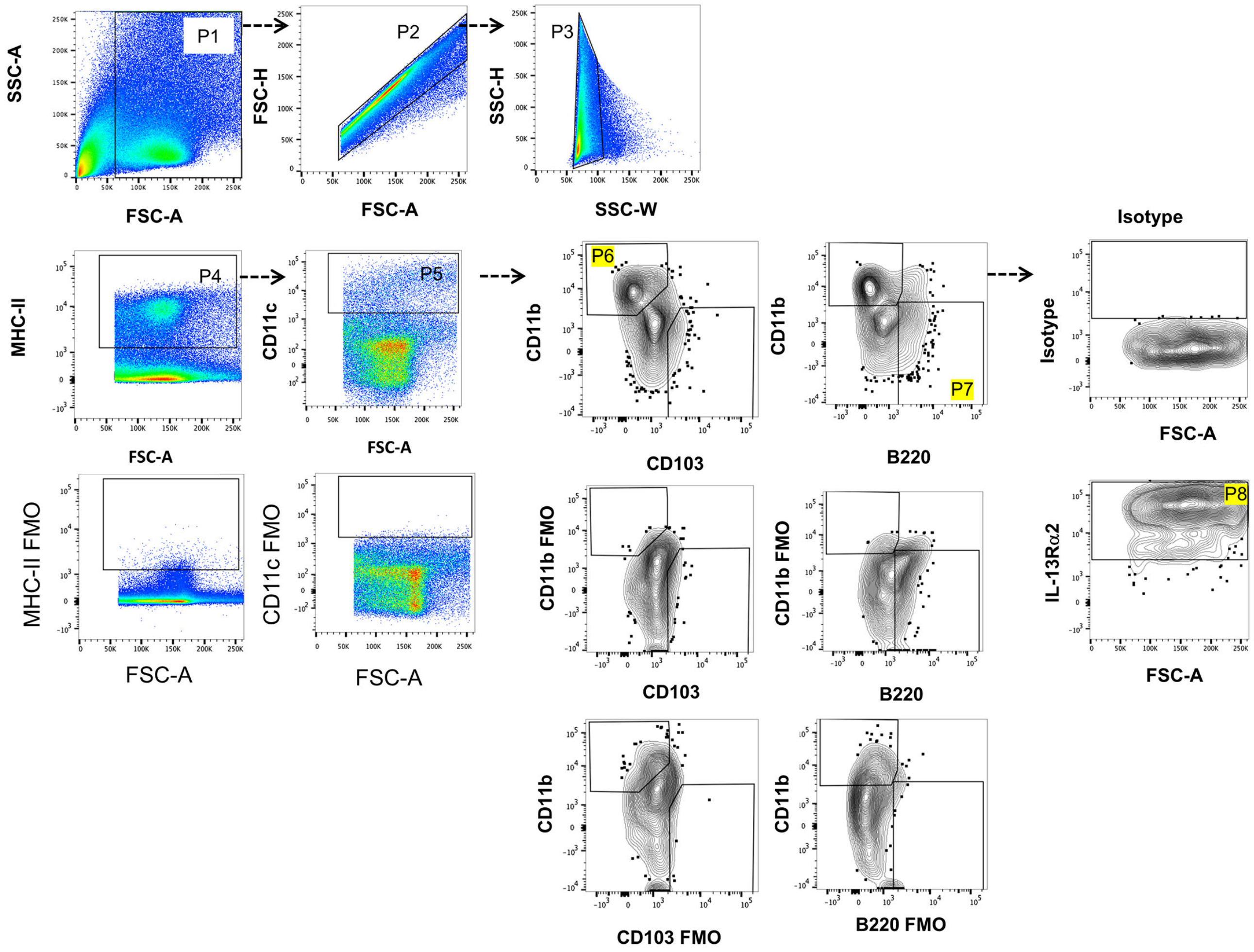

Fig S8. Flow cytometry gating for evaluation of IL-4/IL-13 receptors on lung cDCs and pDCs following i.n. viral vector-based vaccination. Flow cytometry plots show viable cells (P1), followed by single cells based on forward scatter (FSC-H and FSC-A; P2) and side scatter (SSC-H and SSC-W; P3). Single cells were in turn gated on MHC-II^{+} (P4) and CD11c (P5) compared to respective FMO controls. Total DCs (MHC-II^{+} CD11c^{+} - P5) were further gated on CD11b^{+} CD103^{-} cDCs (P6) and CD11b^{-} B220^{+} pDCs (P7) using FMO controls for each marker as indicated. Receptor positive cells (P8) were gated based on isotype controls specific for the time point and viral vector.

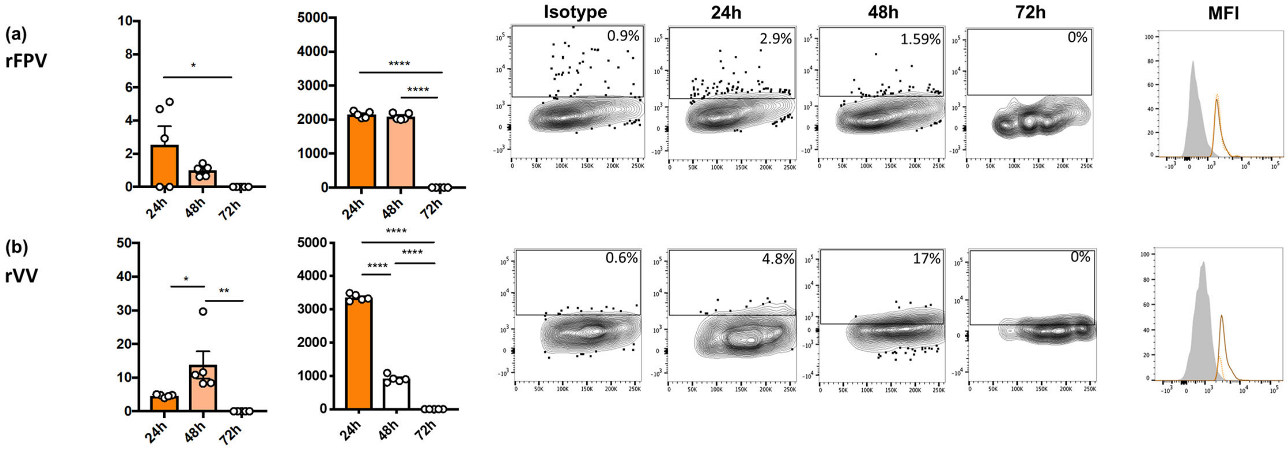

Fig S9. Evaluation of γc expression on lung cDCs at 24, 48 and 72 h following viral vector-based vaccination. BALB/c mice (n=5/ group) were i.n. vaccinated with rFPV or rVV and lungs were harvested at 24, 48 or 72 h post-delivery to evaluate γc expression on lung cDCs using flow cytometry as described in S7 Fig and materials and methods. Bar graphs (left panel) and representative plots (right panel) show percentage of cDCs expressing γc and the corresponding mean fluorescence intensities following **(a)** rFPV and **(b)** rVV vaccination. Histogram plots show γc expression densities at 24 h (solid orange line), 48 h (dotted orange line) and 72 h (tinted orange) compared to the isotype control (solid grey). Error bars represent Standard Error of mean (SEM) and p values were calculated using one-way ANOVA followed by Tukey's multiple comparison test. $*p<0.05$, $**p<0.01$, $***p<0.001$, $****p<0.0001$. Experiments were repeated three times.

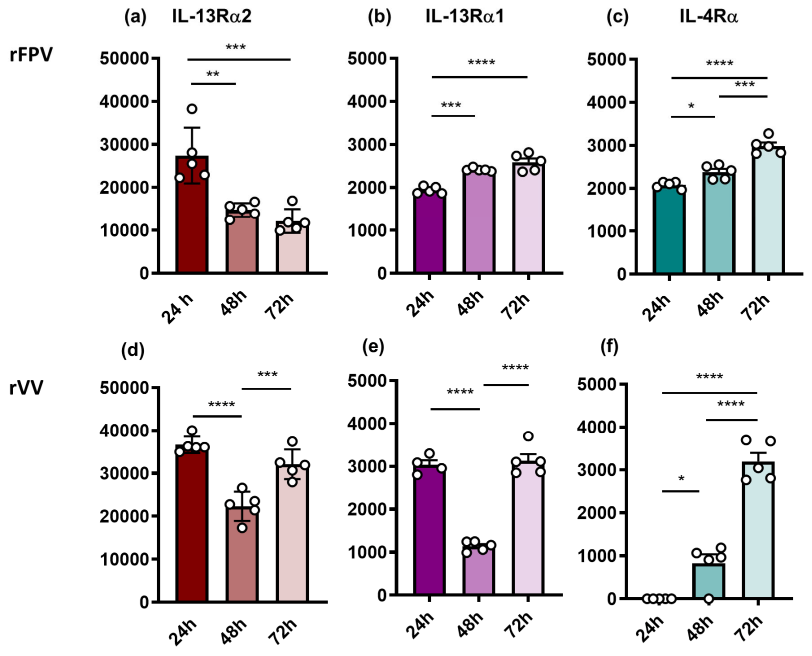

Fig S10. Evaluation of IL-13Rα2, IL-13Rα1, and IL-4Rα receptor densities on lung cDCs at 24, 48 and 72 h following viral vector-based vaccination. BALB/c mice (n=5/group) were i.n. vaccinated with rFPV or rVV and lungs were harvested at 24, 48 or 72 h post-delivery to evaluate IL-13Rα2, IL-13Rα1, and IL-4Rα receptor densities on lung cDCs using flow cytometry as described in S7 Fig and materials and methods. Bar graphs show the mean fluorescence intensities of IL-13Rα2, IL-13Rα1, and IL-4Rα on lung cDCs following **(a)** rFPV and **(b)** rVV vaccination. Error bars represent Standard Error of mean (SEM) and p values were calculated using one-way ANOVA followed by Tukey's multiple comparison test. *p<0.05, **p<0.01, ***p<0.001, ****p<0.0001. Experiments were repeated three times.

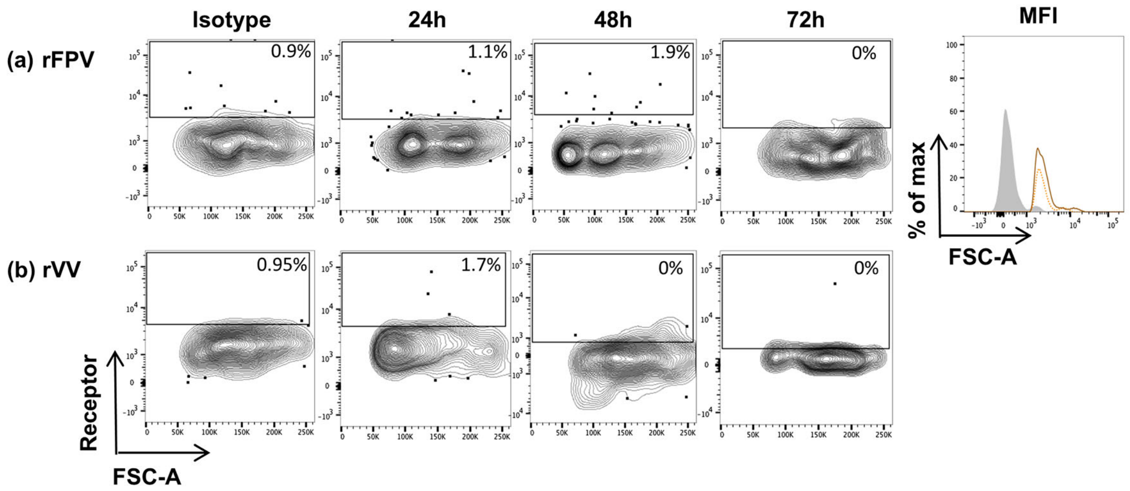

Fig S11. Evaluation of γc expression on lung pDCs at 24, 48 and 72 h following viral vector-based vaccination. BALB/c mice (n=5/ group) were i.n. vaccinated with rFPV or rVV and lungs were harvested at 24, 48 or 72 h post-delivery to evaluate γc expression on lung cDCs using flow cytometry as described in S7 Fig and materials and methods. Representative plots show percentage of $CD11b^-$ $B220^+$ pDCs expressing γc (left panel) following (A) rFPV and (B) rVV vaccination. Flow cytometry histogram plot (right panel) shows γc expression densities at 24 h (solid orange line), 48 h (dotted orange line) and 72 h (tinted orange) compared to the isotype control (solid grey) following rFPV vaccination.

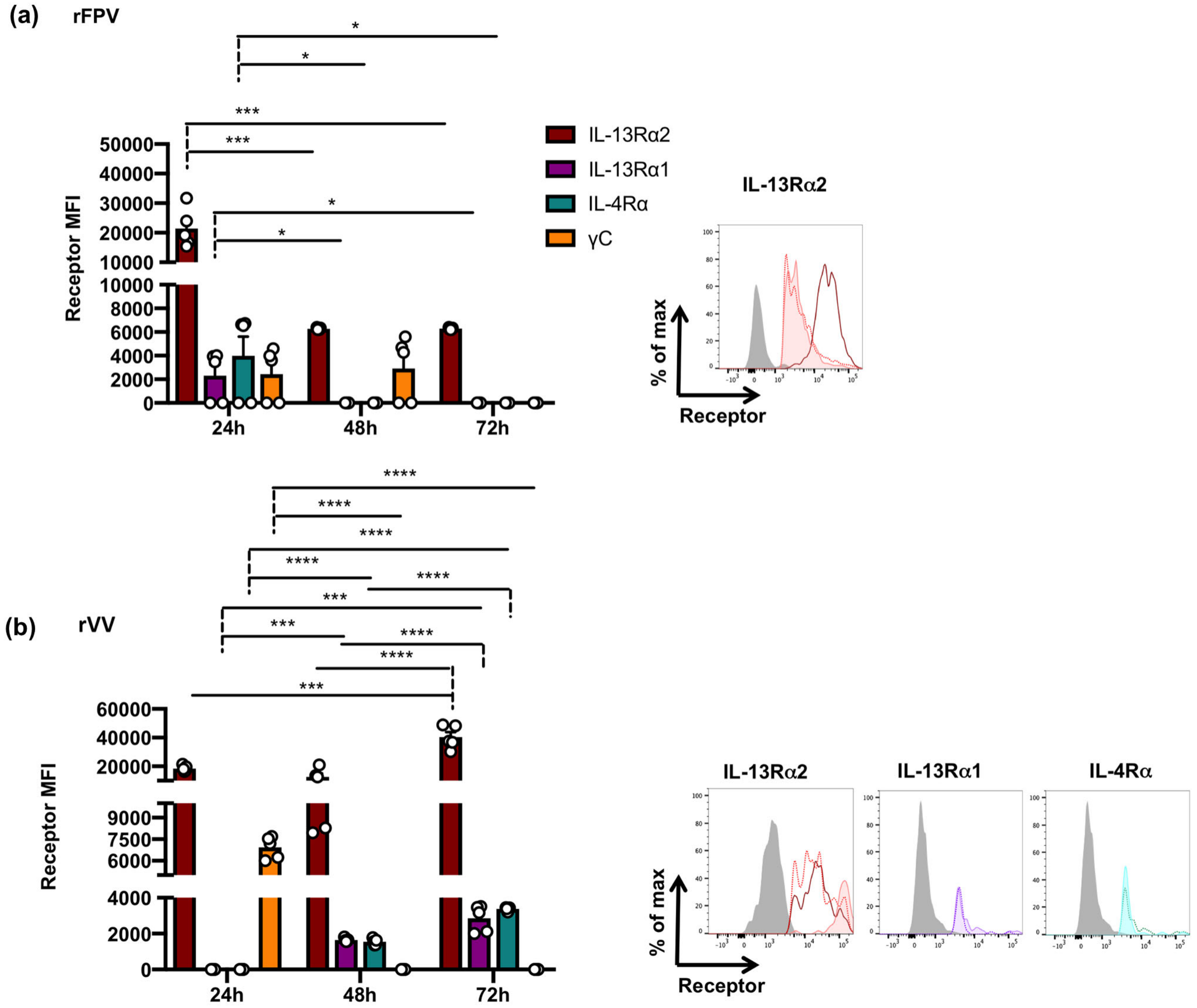

Fig S12. Evaluation of IL-13Rα2, IL-13Rα1, and IL-4Rα receptor densities on lung pDCs at 24, 48 and 72 h following viral vector-based vaccination. BALB/c mice (n=5/group) were i.n. vaccinated with rFPV or rVV and lungs were harvested at 24, 48 or 72 h post-delivery to evaluate IL-13Rα2, IL-13Rα1, and IL-4Rα receptor densities on lung pDCs using flow cytometry as described in S. Fig. 7 and materials and methods. Bar graphs show the mean fluorescence intensities of IL-13Rα2, IL-13Rα1, and IL-4Rα on lung pDCs following (a) rFPV and (b) rVV vaccination. Error bars represent Standard Error of mean (SEM) and p values were calculated using one-way ANOVA followed by Tukey's multiple comparison test. *$p<0.05$, **$p<0.01$, ***$p<0.001$, ****$p<0.0001$. Experiments were repeated three times.

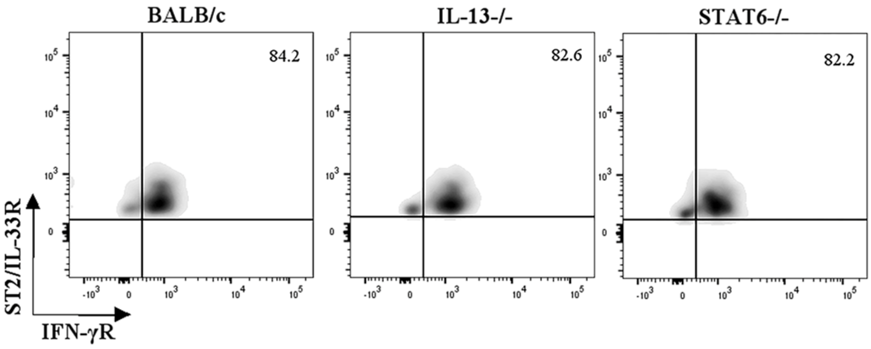

Fig S13. Evaluation of IFN-γR on lung ST2/IL-33R$^+$ ILC2, 24 h post FPV-HIV vector vaccination. WT BALB/c, STAT6$^{-/-}$ and IL-13$^{-/-}$ mice on the BALB/c background were immunized intranasally with unadjuvanted FPV-HIV vaccine. Lung ILC2 were gated as CD45$^+$ FSC^{low} SSC^{low} lineage$^-$ IL-33R/ST2$^+$ cells. The FACS plot indicated IFN-γR expression on ST2/IL-33R$^+$ ILC2 24 h post intranasal FPV-HIV vaccination.

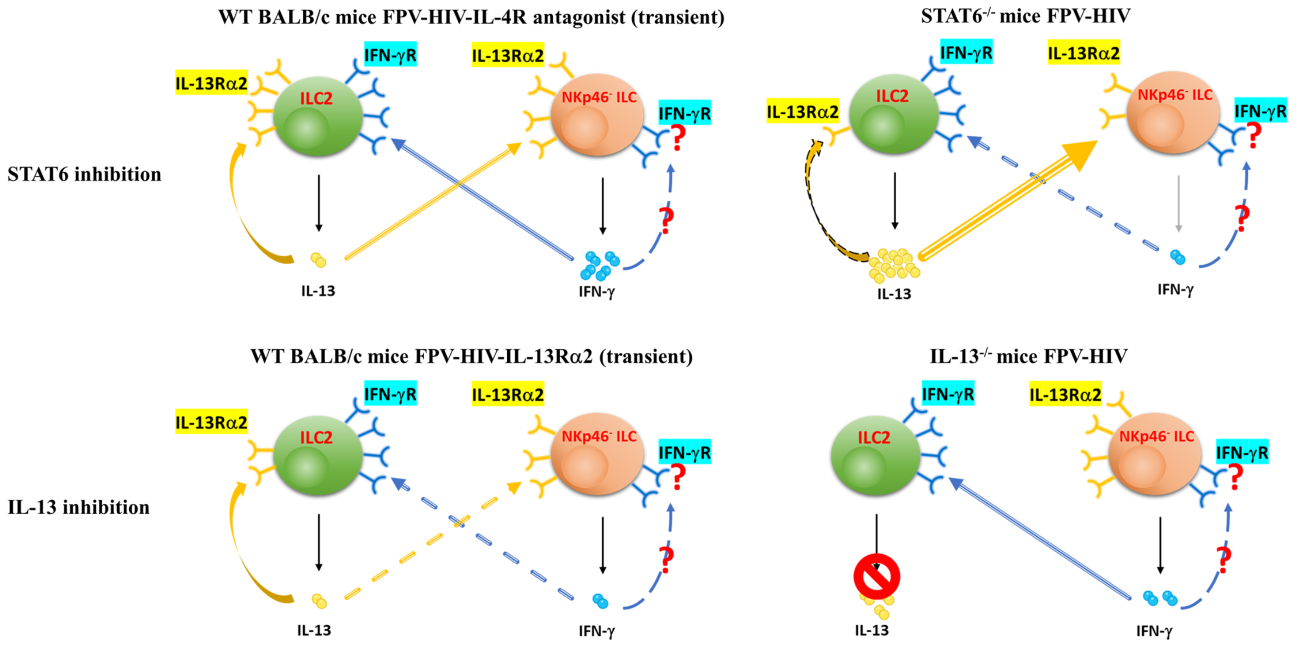

Fig S14. Schematic diagram showing the proposed co-regulation of ILC2-derived IL-13 and inter-regulation of ILC2 and ILC1/ILC3 under permanent vs transient STAT6 and IL-13 inhibition conditions. Under STAT6$^{-/-}$ scenario, high IL-13 expressed by ILC2 is sequestered by IL-13Rα2 on NKp46^{-} ILC1/ILC3 resulting in reduced IFN-γ expression, unlike transient STAT6 inhibition condition. Under transient IL-13 inhibition scenario, FPV-HIV-IL-13Rα2 vaccine sequesters IL-13 in the cell milieu thus no IL-13 signalling occurs via IL-13Rα2 on NKp46^{-} ILC1/ILC3 resulting in reduced IFN-γ expression. Note that the solid arrows indicate regulation/activity occur, and dotted arrows indicate regulation/activity may or may not occur.
